# Solvent-Driven Variation in the Determination of Antioxidant Capacity and Oxidative Stress Indicators of Extra Virgin Olive Oils from the Aegean Region

**DOI:** 10.3390/foods15122092

**Published:** 2026-06-10

**Authors:** Aslıhan İlayda İlhan, Suzan Yalçın, Sıddika Songül Yalçın

**Affiliations:** 1 Institute of Health Sciences, Selcuk University, Konya 42130, Türkiye; aslihanilayda@hotmail.com; 2 Department of Food Hygiene and Technology, Faculty of Veterinary Medicine, Selcuk University, Konya 42003, Türkiye; 3 Division of Social Pediatrics, Department of Pediatrics, Faculty of Medicine, Hacettepe University, Ankara 06230, Türkiye; siyalcin@hacettepe.edu.tr

**Keywords:** extra virgin olive oil, solvent extraction, phenolic compounds, oxidative stress index, principal component analysis

## Abstract

This study investigated the influence of different extraction solvents (ethanol and methanol) on antioxidant and oxidative stress parameters in 55 extra virgin olive oil samples obtained from various producers in the Aegean region of Türkiye. In this context, DPPH radical scavenging activity, total phenolic content (TPC), total antioxidant status (TAS), total oxidant status (TOS), and oxidative stress index (OSI) were determined in both extracts. Ethanol extracts showed higher TPC and DPPH radical scavenging activity, with mean DPPH values of 24.48% and 15.42% for ethanol and methanol extracts, respectively. Similarly, TOS and OSI values were higher in ethanol extracts, whereas TAS values were higher in methanol extracts. Correlation analysis revealed significant negative relationships between antioxidant parameters (TPC and TAS) and OSI, indicating an inverse association between antioxidant capacity and oxidative stress. Principal component analysis (PCA) demonstrated a distinct separation between antioxidant and oxidant variables. Bland–Altman analysis further confirmed systematic differences between extraction methods. Overall, the findings indicate that the extraction solvent is a critical factor in the determination of antioxidant and oxidative stress parameters in olive oil. Ethanol provides a higher phenolic content and radical scavenging activity, whereas methanol appears more effective in assessing total antioxidant capacity. The variability among samples reflects differences in production, processing, and storage conditions.

## 1. Introduction

Extra virgin olive oil (EVOO) is widely recognized as a key component of the Mediterranean diet and has attracted significant attention due to its nutritional value and potential health benefits. Its positive biological effects are largely attributed to the abundance of monounsaturated fatty acids, mainly oleic acid, together with several minor bioactive constituents such as phenolic compounds, tocopherols, phytosterols, carotenoids, and chlorophyll pigments. Among these compounds, phenolics—including hydroxytyrosol and oleuropein—play a particularly important role in enhancing the antioxidant properties and oxidative stability of olive oil [1,2].

Numerous studies have demonstrated a significant correlation between total phenolic content (TPC) and antioxidant potential of EVOO. Phenolic compounds have been shown to contribute significantly to oxidative protection in biological systems [3]. Similarly, olive oils containing greater amounts of phenolic compounds generally exhibit stronger antioxidant capacity and enhanced resistance to oxidative deterioration [4,5]. Gorinstein et al. [4] reported values of 4.6 mg/kg total polyphenols, 29.4% DPPH radical scavenging activity (RSA), and 2.64 mmol TE/kg total antioxidant activity in EVOO samples. Their study also revealed significant positive relationships between total phenolic content and several antioxidant parameters, including DPPH RSA (R^2^ = 0.986), total antioxidant activity (R^2^ = 0.9197), and β-carotene bleaching assay results (R^2^ = 0.9958). In addition, Türk Baydır [6] reported that extra virgin olive oil exhibited the highest antioxidant activity (DPPH RSA = 68.143%) and oxidative stability among edible oils. Güzel et al. [5] also demonstrated that extra virgin olive oil had the highest total antioxidant capacity (TAC) and TPC, and that TAC values were positively correlated with phenolic content.

In recent years, oxidative stress-related indices such as total oxidant status (TOS) and oxidative stress index (OSI) have attracted increasing attention as complementary indicators for evaluating oxidative balance in food systems. These parameters provide additional information beyond conventional antioxidant assays by reflecting the overall pro-oxidant burden of complex matrices. Natural antioxidants provide protection via multiple pathways, such as neutralizing free radicals, binding metal ions, lowering localized oxygen levels, and breaking down peroxide compounds [7]. Previous studies have indicated that the contribution of polyphenols to oxidative stability and antioxidant activity in virgin olive oil is greater than that of other antioxidant compounds [7,8]. Del Carlo et al. [7] further demonstrated that oxidative stability in EVOO depends not only on antioxidant compounds but also on the equilibrium established among oxidizing and antioxidative constituents present in the complex polyphenolic matrix of olive oil.

Extraction methodology also plays a critical role in the analysis of phenolic compounds and antioxidative properties sample matrix characteristics, and compound–solvent interactions directly affect extraction efficiency. Extraction efficiency of phenolic compounds is strongly influenced by solvent polarity, solvent composition, and physicochemical interactions between phenolic compounds and the sample matrix. Polar organic solvents, particularly methanol and ethanol, are among the most widely used extraction solvents for phenolic compounds because they enhance the solubilization of compounds with different polarities and facilitate disruption of phenolic–matrix interactions through hydrogen-bonding mechanisms [9,10,11,12]. Previous studies demonstrated that aqueous alcohol mixtures generally provide better extraction performance than pure water due to enhanced solubilization and recovery of phenolic compounds from complex matrices [11,13]. In olive leaf and olive-derived matrices, hydroalcoholic systems containing 50–80% methanol or ethanol are widely used for improving the recovery of phenolic compounds and antioxidant activity [13,14]. Differences between methanol–water and ethanol–water extraction systems may be associated with variations in solvent polarity and hydrogen-bonding interactions, which can influence the solubilization, recovery, and antioxidant-related properties of phenolic compounds [10,11].

Given the complex biochemical composition of EVOO and the multidimensional relationships among antioxidant and oxidant parameters, multivariate approaches, including principal component analysis (PCA), are being increasingly widely applied for data interpretation. PCA enables dimensionality reduction and facilitates the identification of relationships among multiple biochemical variables, providing a more comprehensive evaluation of olive oil quality. Accordingly, this study was designed to examine how ethanol and methanol extraction influence antioxidant indicators (TPC, DPPH, TAS) as well as oxidant-related parameters (TOS and OSI) in extra virgin olive oil samples obtained from various producers and marketed in the Aegean region of Türkiye. Furthermore, multivariate statistical methods were employed to explore the interrelationships among these variables and to gain deeper insights into how the choice of extraction solvent affects the oxidative and antioxidant characteristics of the samples.

## 2. Materials and Methods

### 2.1. Materials

In the present work, 55 extra virgin olive oil samples obtained from different producers and marketed in the Aegean Region were used as the study material. The samples were randomly selected from commercially available products and stored in their original packaging under appropriate conditions, protected from light and heat until analysis. All analyses were performed without any pre-treatment of the samples.

### 2.2. Methods

#### 2.2.1. Extraction of Samples

Methanol and ethanol extracts of the olive oil samples were prepared. Hydroalcoholic solvent systems (80:20, *v*/*v*) were selected based on previous studies reporting effective recovery of phenolic compounds from olive-derived matrices using aqueous methanol and ethanol extraction systems [15,16]. Such hydroalcoholic extraction approaches are among the most commonly used conventional extraction systems for phenolic compound recovery [13].

Preparation of methanol extracts: Approximately 0.5 g of olive oil was accurately weighed into 15 mL glass tubes. The lipid fraction was first dissolved by adding 5 mL of *n*-hexane, followed by vortex mixing for 1 min. Subsequently, 5 mL of a methanol/distilled water solution (80:20, *v*/*v*) was introduced. The mixture was then vortexed for 2 min to promote the migration of phenolic and antioxidant compounds into the polar phase. Afterward, the mixtures were subjected to centrifugation at 4000 rpm for a duration of 10 min. Following centrifugation, the upper hexane layer containing lipophilic components was carefully discarded. The lower methanol/water phase (polar extract) was used for TPC, DPPH, TAS, and TOS analyses.

Preparation of ethanol extracts: The same procedure described above was applied, except that methanol/distilled water (80:20, *v*/*v*) was replaced with ethanol/distilled water (80:20, *v*/*v*). The resulting ethanol/water phase was used for TPC, DPPH, TAS, and TOS analyses.

#### 2.2.2. Determination of Total Phenolic Content

TPC in the olive oil samples was evaluated using the Folin–Ciocalteu colorimetric assay [17,18]. In short, 100 µL of the extract (either methanolic or ethanolic) was combined with 500 µL of distilled water, followed by the addition of 100 µL of Folin–Ciocalteu reagent and 1000 µL of 7% sodium carbonate solution. An additional 500 µL of distilled water was then introduced, and the reaction mixture was kept in the dark for 90 min to allow color development. Absorbance was subsequently recorded at 760 nm. Gallic acid served as the calibration standard. A stock solution of gallic acid (20 mg/mL) of gallic acid was prepared, and working standards ranging from 0.05 to 0.75 mg/mL were obtained through serial dilution in methanol for the methanolic extracts. The calibration plot (y = 4.950x; where y represents absorbance and x represents gallic acid concentration) exhibited strong linearity (R^2^ = 0.996). For ethanol extracts, serial dilutions (0.0125–0.25 mg/mL) were prepared in ethanol. The calibration curve (y = 6.432x; R^2^ = 0.996) also demonstrated high linearity. Results were expressed as milligrams of gallic acid equivalents per kilogram of sample (mg GAE/kg).

#### 2.2.3. Determination of DPPH Radical Scavenging Activity

DPPH RSA was evaluated following the procedure described by Blois [19], as indicated by Onbaşılar et al. [17]. In brief, 200 µL of the extract was combined with 800 µL of distilled water, after which 1 mL of a 0.2 mM DPPH solution prepared in methanol was added. The mixture was vortexed and then kept in the dark at room temperature for 30 min to allow the reaction to proceed. Measurement of absorbance was performed at 517 nm. The DPPH RSA percentage was determined using the equation given below:

DPPH RSA (%) = [1 − (sample absorbance/control absorbance)] × 100

Control absorbance values were measured separately for methanol and ethanol.

#### 2.2.4. Determination of Total Antioxidant Status (TAS)

TAS of olive oil samples was determined using a colorimetric method as reported by Onbaşılar et al. [20] with a commercial assay kit (Rel Assay Diagnostics, Cat. No: RL0017, Gaziantep, Türkiye). Analyses were performed using an autoanalyzer (Mindray BS-400, Shenzhen, China) according to the manufacturer’s instructions. The method is based on the ability of antioxidants in the sample to suppress free radical cations. Results were quantified using Trolox as the calibration standard and reported as millimoles of Trolox equivalents per kilogram (mmol TE/kg).

#### 2.2.5. Determination of Total Oxidant Status (TOS)

TOS was evaluated according to the procedure described by Onbaşılar et al. [20], which relies on the conversion of ferrous ions in the *o*-dianisidine complex into ferric ions through oxidation. The measurements were performed using a commercial TOS assay kit (Rel Assay Diagnostics, Cat. No: RL0024, Gaziantep, Türkiye) with an autoanalyzer (Mindray BS-400, Shenzhen, China). Hydrogen peroxide was used as the calibration standard, and the results were expressed as micromoles of hydrogen peroxide equivalents per kilogram (µmol H_2_O_2_ eq/kg).

#### 2.2.6. Calculation of Oxidative Stress Index (OSI)

OSI was calculated to evaluate the oxidative balance of the samples, using the ratio of TOS to TAS as reported by Onbaşılar et al. [20]:

OSI = TOS (µmol H_2_O_2_ equivalent/kg) × 100/TAS (µmol Trolox equivalent/kg)

This parameter reflects the balance between oxidizing and antioxidant components in the samples in a quantitative manner.

### 2.3. Statistical Analysis

Statistical evaluation was performed with R software (version 4.5.1; R Foundation for Statistical Computing, Vienna, Austria) together with IBM SPSS Statistics 23.0 (IBM Corp., Armonk, NY, USA). A probability value below 0.05 was considered statistically significant. Data distribution was analyzed using the Shapiro–Wilk test, while skewness and kurtosis values were also reviewed to support the assessment of normality (Table 1). The results showed that the majority of variables deviated from a normal distribution (*p* < 0.05). For this reason, the median was used to describe central tendency in the dataset. Relationships between biochemical parameters and agreement between extraction methods were evaluated using Spearman correlation analysis, and results were visualized using heatmaps. Differences between the two extraction methods (ethanol and methanol) were analyzed using the Wilcoxon signed-rank test for paired samples. To reduce the likelihood of Type I errors arising from multiple testing, *p*-values were adjusted using the Benjamini–Hochberg false discovery rate (FDR) procedure. Additionally, Bonferroni correction results were reported for validation.

Percentage changes between methods were calculated using the following formula based on median values:

(Methanol value − Ethanol value) × 100/Ethanol value

PCA was applied to visualize the overall structure of antioxidant–oxidant parameters and differences between methods. Prior to PCA, variables were standardized using z-scores. Bland–Altman plots were constructed to evaluate systematic differences, bias, and 95% limits of agreement between methods. Radar plots were generated using normalized data to compare the characteristic metabolic profiles (fingerprints) of extraction methods.

Data analysis and visualization were conducted using the following R packages: ggplot2 (for graphics), FactoMineR (for PCA), fmsb (for radar plots), and stats (for non-parametric tests).

## 3. Results

Descriptive statistics of TPC, DPPH RSA, TAS, TOS, and OSI of olive oil samples extracted with ethanol and methanol are presented in Table 2. When TPC values were examined, the mean value was found to be 138.1 mg GAE/kg for ethanol extracts and 123.0 mg GAE/kg for methanol extracts. The minimum–maximum ranges were 50.2–297.0 mg GAE/kg for ethanol and 10.1–341.4 mg GAE/kg for methanol, indicating a wide distribution of phenolic content in both solvents.

Regarding DPPH RSA, the mean percentage was 24.48% for ethanol extracts and 15.42% for methanol extracts. The maximum inhibition reached 38.77% in ethanol extracts, whereas it remained lower in methanol extracts (26.14%), suggesting that ethanol extracts exhibited higher antioxidant activity.

TAS values were higher in methanol extracts (3.2 mmol TE/kg) compared to ethanol extracts (2.8 mmol TE/kg), with the maximum TAS value reaching 7.8 mmol TE/kg in methanol. In contrast, TOS values were higher in ethanol extracts (mean: 84.6 µmol H_2_O_2_ Eq/kg) than in methanol extracts (70.5 µmol H_2_O_2_ Eq/kg), although the maximum values were similar in both solvents. When OSI values were evaluated, the mean was 3.51 for ethanol extracts and 2.51 for methanol extracts, indicating lower oxidative stress in methanol extracts.

The type of solvent had a significant effect on antioxidant and oxidant parameters. Ethanol extracts showed higher DPPH RSA activity and TOS values, whereas methanol extracts exhibited higher TAS and lower OSI values.

**Figure 1:**
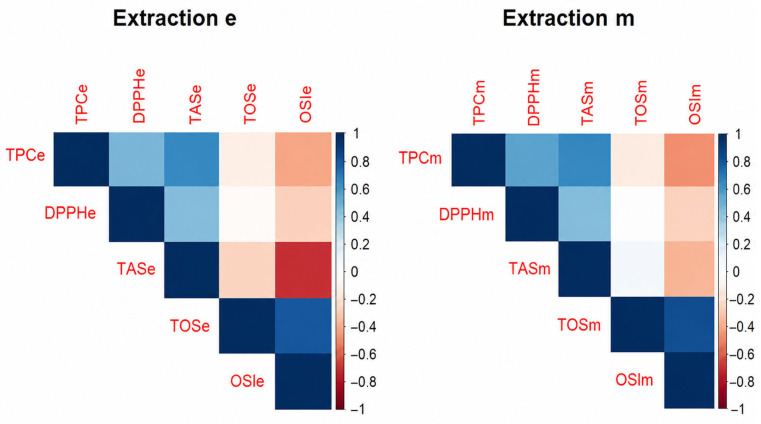
Correlation heatmaps showing the relationships between antioxidant and oxidant parameters according to different solvents. Dark blue regions represent positive correlations, whereas red/orange regions indicate negative correlations; e: ethanol; m: methanol; TPC: total phenolic content; DPPH: 2,2-diphenyl-1-picrylhydrazyl; TOS: total oxidant status; TAS: total antioxidant status; OSI: oxidative stress index.

Correlation analysis revealed a biochemically consistent balance between antioxidant and oxidative stress markers (Table 3, Figure 1). Oxidative stress markers showed strong positive correlations among themselves, with the highest correlation observed between TOS and OSI (dark blue regions in Figure 1). Similarly, a positive relationship was found between antioxidant parameters TPC and TAS. In contrast, antioxidant parameters (TPC, TAS) were negatively correlated with OSI, confirming the antioxidant–oxidant balance in samples across both extraction methods (red/orange regions in Figure 1). DPPH exhibited positive correlations with TPC and TAS in both extracts.

Spearman correlation analysis performed to assess agreement between solvents demonstrated high positive relationships for TPC (r = 0.735), TOS (r = 0.706), and OSI (r = 0.807) between ethanol and methanol extractions (Table 4).

The impact of different extraction solvents on antioxidant and oxidant-related parameters was additionally examined through paired comparison analyses (Table 5). Median-based comparisons indicated that TPC, DPPH RSA, TOS, and OSI values were higher when ethanol was used as the solvent, whereas TAS values were slightly higher with methanol. Compared to methanol, ethanol increased TPC by 17.97%, DPPH RSA by 37.25%, TOS by 40.62%, and OSI by 43.87%. In contrast, TAS showed a modest increase of 4.01% with methanol compared to ethanol. After applying the Benjamini–Hochberg (FDR) correction for multiple comparisons, all parameters (TPC, DPPH RSA, TAS, TOS, OSI) remained statistically significant. However, following Bonferroni correction, only DPPH RSA, TOS, and OSI differences remained significant (*p* ≤ 0.001), while TPC and TAS differences were no longer statistically significant (*p* > 0.05).

**Figure 2:**
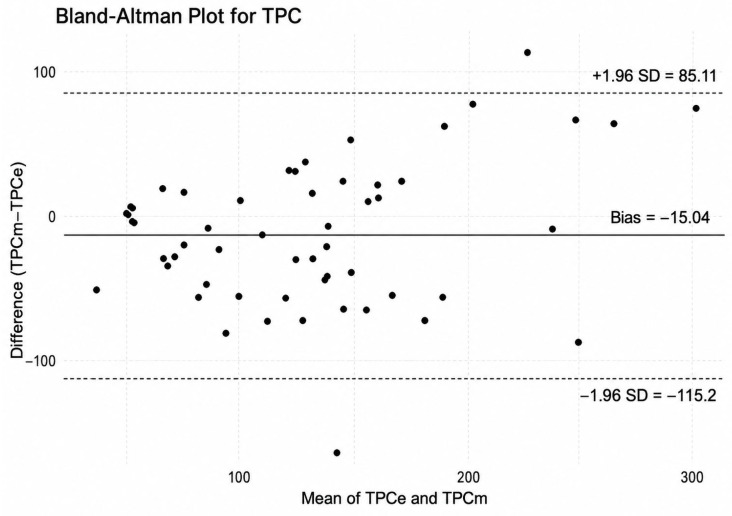
Bland–Altman agreement plots for TPC in ethanol and methanol extracts. The solid line represents the mean difference (bias), while the dashed lines indicate the 95% limits of agreement (±1.96 SD).

**Figure 3:**
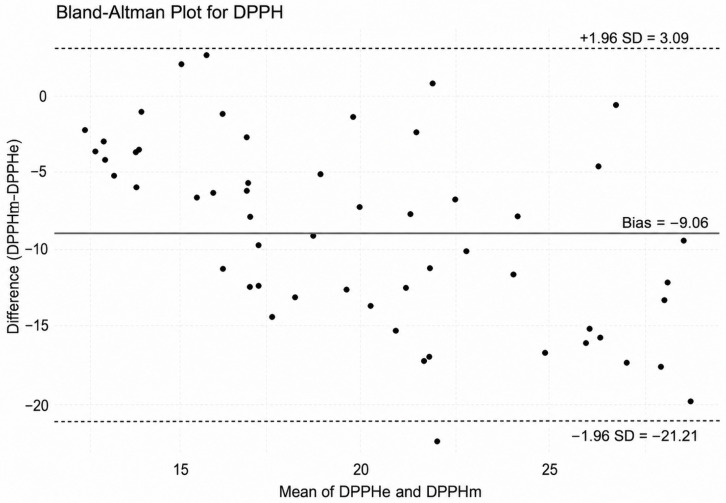
Bland–Altman agreement plots for DPPH in ethanol and methanol extracts. The solid line represents the mean difference (bias), while the dashed lines indicate the 95% limits of agreement (±1.96 SD).

**Figure 4:**
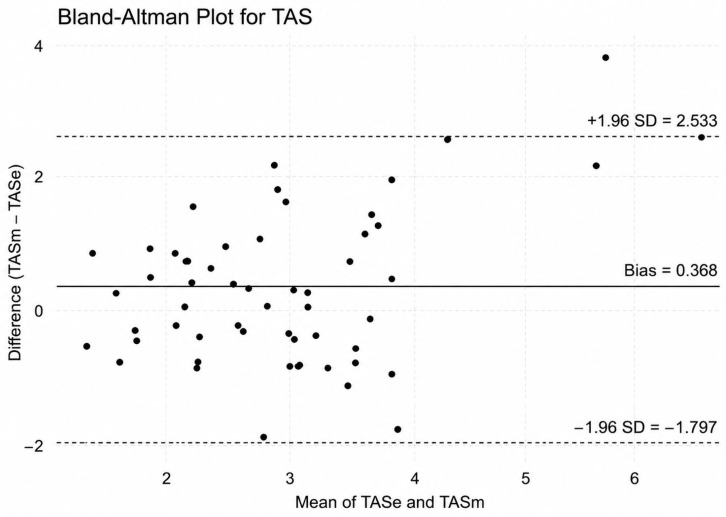
Bland–Altman agreement plots for TAS in ethanol and methanol extracts. The solid line represents the mean difference (bias), while the dashed lines indicate the 95% limits of agreement (±1.96 SD).

**Figure 5:**
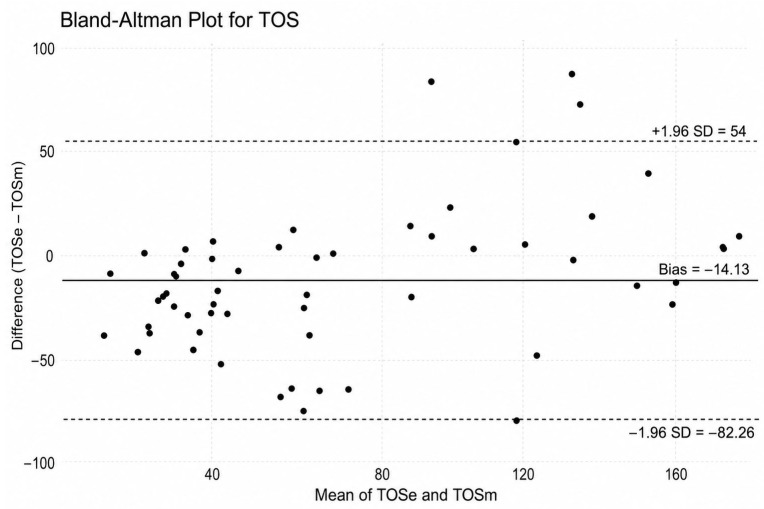
Bland–Altman agreement plots for TOS in ethanol and methanol extracts. The solid line represents the mean difference (bias), while the dashed lines indicate the 95% limits of agreement (±1.96 SD).

**Figure 6:**
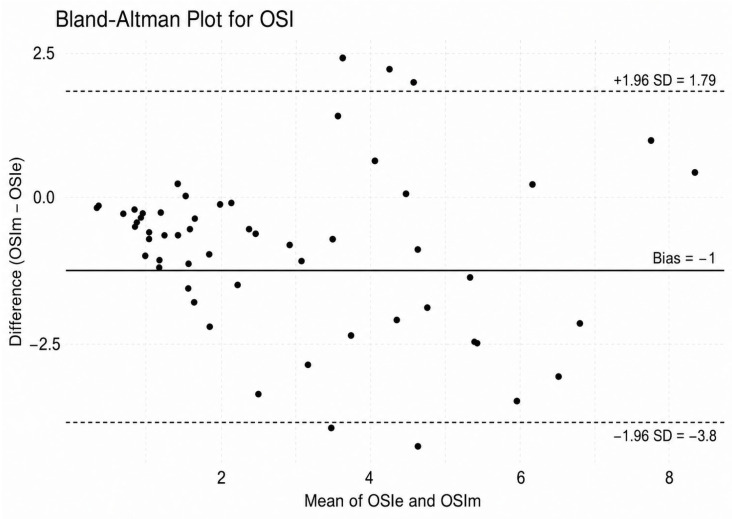
Bland–Altman agreement plots for OSI in ethanol and methanol extracts. The solid line represents the mean difference (bias), while the dashed lines indicate the 95% limits of agreement (±1.96 SD).

Bland–Altman analysis (Figure 2, Figure 3, Figure 4, Figure 5 and Figure 6) revealed systematic differences between solvents for most parameters. Negative bias (mean difference) values were observed for TPC, DPPH RSA, TOS, and OSI (Table 6), indicating that ethanol generally yielded higher measurements than methanol. The mean difference for TPC was −15.04 (limits of agreement: −115.20 to 85.11), showing greater variability compared with other parameters. TAS showed a small positive bias (0.37) with limits of agreement ranging from −1.80 to 2.53, indicating close agreement between the two extraction methods. Most data points were within the 95% limits of agreement (±1.96 SD), and no clear proportional bias (slope or funnel pattern) was observed, indicating that the differences between methods were largely independent of measurement magnitude.

**Figure 7:**
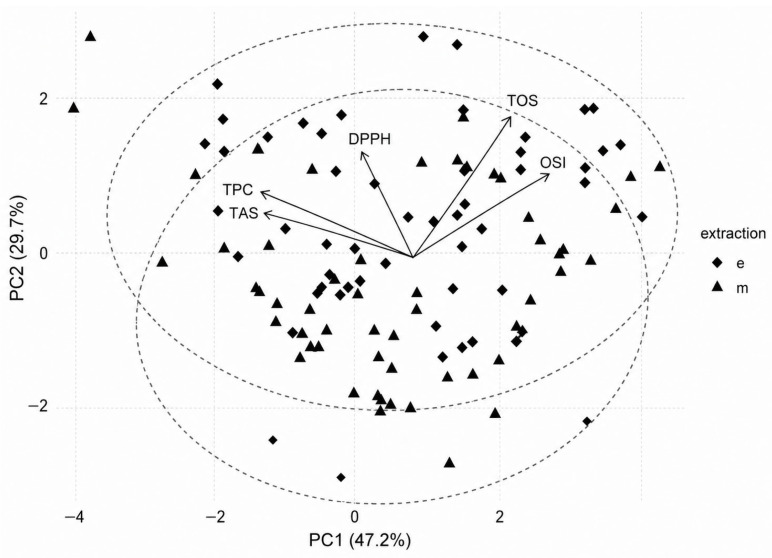
PCA biplot showing score distribution. The figure illustrates the separation tendency of different solvents according to their overall biochemical profiles. Vectors (TPC, DPPH, TAS, TOS, OSI) represent the loading patterns of variables on the principal components (PC1 and PC2) and their contributions to the variance. DPPH: 2,2-diphenyl-1-picrylhydrazyl; TPC: total phenolic content; TOS: total oxidant status; TAS: total antioxidant status; OSI: oxidative stress index.

PCA revealed the multivariate structure of antioxidant and oxidant parameters (Table 7). The highest positive loading on PC1 was observed for OSI (0.547), while the highest negative loadings were found for TPC (−0.491) and TAS (−0.487), indicating that PC1 represents a contrast between oxidative stress and antioxidant capacity. On PC2, the most influential parameters were TOS (0.600) and DPPH (0.475). OSI was the dominant contributor to PC1, explaining approximately 30% of the variance. The first two principal components together explained 76.9% of the total variance (PC1: 47.2%, PC2: 29.7%) (Figure 7). The PCA biplot indicated a tendency for separation between ethanol and methanol extracts, suggesting that the extraction methods produce distinct biochemical profiles. Oxidant variables (TOS, OSI) and antioxidant parameters (TPC, TAS, DPPH) were positioned in opposite directions along the principal component axes, contributing to this separation. Notably, TAS, TPC, and DPPH showed similar orientations, whereas TOS and OSI diverged along the PC1 axis.

**Figure 8:**
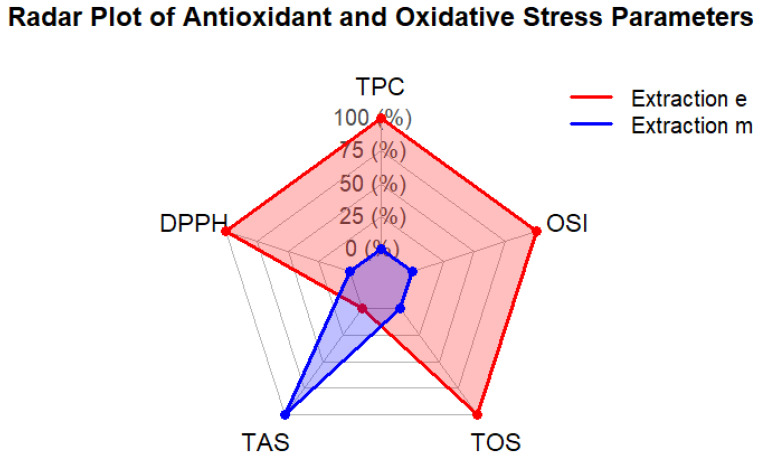
Comparison of normalized antioxidant and oxidant profiles of ethanol and methanol extracts using a radar chart. e: ethanol; m: methanol; DPPH: 2,2-diphenyl-1-picrylhydrazyl; TPC: total phenolic content; TOS: total oxidant status; TAS: total antioxidant status; OSI: oxidative stress index.

Radar plots generated from normalized data (Figure 8) clearly distinguished the antioxidant and oxidant profiles of the two solvents. Ethanol (red area) exhibited higher values for TPC, DPPH RSA, TOS, and OSI, resulting in a generally larger radar area. Methanol (blue area), in comparison, showed a higher value only for TAS. This pattern indicates that ethanol is associated with higher extraction of both antioxidant and oxidant-related parameters.

## 4. Discussion

In this study, ethanol and methanol were used as extraction solvents to assess their influence on antioxidant and oxidative stress-related parameters in extra virgin olive oil samples obtained from the Aegean Region. The results indicate that the choice of solvent plays a crucial role in shaping both the measured antioxidant potential and oxidant levels of the samples. It is widely recognized that dietary intake of oils rich in natural antioxidants is linked to a lower risk of cardiovascular disorders [21].

Phenolic compounds can inhibit oxidation through various mechanisms, including radical scavenging, hydrogen atom transfer, and metal chelation [22]. The TPC findings indicated that ethanol-based extracts yielded higher phenolic content values than those obtained with methanol. Previous studies have reported that TPC values in olive oils generally range between 50 and 500 mg GAE/kg, depending on cultivar, harvest time, and production conditions [22,23], which aligns with the results obtained in the present study. In contrast, Hrncirik and Fritsche [24] reported that concentrations of polar phenolic fractions in premium-quality olive oils can vary between 200 and 1500 mg/kg. Similarly, Salvador et al. [25] found average total phenolic levels of 147 mg/kg (ranging from 19 to 380 mg/kg) in commercial Cornicabra extra virgin olive oils, while Abencor-extracted samples showed higher values of 367 mg/kg (180–614 mg/kg).

In this study, prior n-hexane treatment removed most of the lipophilic fraction, and analyses were primarily focused on polar phenolic compounds. Therefore, the higher TPC values observed in ethanol extracts may be attributed to the selective extraction of phenolic compounds with different polarities, since solvent polarity and solvent–matrix interactions significantly influence extraction efficiency and phenolic composition [22,26,27]. Additionally, cultivar (especially Koroneiki), harvest time, climatic conditions, and production techniques are known to significantly affect phenolic composition [28,29].

In this study, the ethanol extracts exhibited a DPPH RSA value of 24.48%, while the methanol extracts showed a lower activity of 15.42%. The higher DPPH RSA observed in ethanol extracts may be associated with the extraction of antioxidant-related compounds, particularly phenolic constituents known for their free radical scavenging activity. The antioxidant effectiveness of extra virgin olive oil is affected by the quantity and profile of naturally occurring active molecules, including both phenolic and non-phenolic constituents. In addition, these compounds may vary according to olive variety and the maturity level of the fruit during harvest [30,31,32].

Consistent with these findings, Sicari [23] reported DPPH RSA ranging from 18.33% to 36.85% in EVOOs obtained from three different Calabrian olive cultivars in Italy, with statistically significant differences among samples. Seydou et al. [33] reported DPPH RSA values of 94%, 92%, 91%, and 28% for Kilis yağlık, İzmir sofralık, Ayvalık, and Tavşan yüreği olive oils, respectively, highlighting dose-dependent antioxidant activity. It was also noted that Tavşan yüreği oil exhibited considerably lower antioxidant activity compared to the others. Bouarroudj et al. [34] reported that wild olive (oleaster) extracts exhibited higher antioxidant capacity than cultivated oils, and that this activity remained more stable during advanced ripening stages. Similarly, Faci et al. [31] found a significant decline in DPPH RSA during ripening, with reductions exceeding 30% in Chemlal and Limli cultivars and approximately 18% in Oleaster extracts. These findings indicate that early-harvest oils possess higher RSA. In the present study, the evaluated extra virgin olive oils are likely derived from late-harvest fruits.

It has also been shown that the antioxidant activity of olive oils depends on cultivar, geographical origin, and degree of ripening [33,35]. Furthermore, the positive relationships between TPC and DPPH RSA support the key role of phenolic compounds in antioxidant activity [23,33].

In this study, TAS values were 2.8 mmol TE/kg for ethanol extracts and 3.2 mmol TE/kg for methanol extracts. Öğüt [36] reported a lower value of 1.279 mM TE/L in olive oils from Aydın using 50% methanol without hexane extraction. Pellegrini et al. [37] reported total antioxidant activity values ranging from 0.72 to 1.06 mmol TE/kg in commercial olive oils, from 1.53 to 2.69 mmol TE/kg in extra virgin olive oils, and around 0.61 mmol TE/kg in refined oils. Total antioxidant capacity is considered a simple and effective method reflecting the overall antioxidant content of a sample [37].

The higher TAS values observed in methanol extracts are noteworthy. This may be explained by the removal of the lipophilic fraction via n-hexane and the higher polarity of methanol, which may favor the extraction of relatively polar phenolic compounds. Xu and Chang [27] reported that solvents with different polarities significantly affect both extracted compounds and antioxidant activity. The efficiency of methanol in extracting low-molecular-weight phenolics has also been emphasized [9]. Condelli et al. [22] further stated that antioxidant capacity depends not only on total phenolic content but also on phenolic structure and solubility, making solvent-dependent variation expected.

Higher TOS and OSI values in ethanol extracts indicate that this solvent extracts not only antioxidant compounds but also oxidant-related components more efficiently. Since OSI is based on the TOS/TAS ratio, this finding is consistent with increased oxidative load. Lower OSI values in methanol extracts may indicate lower co-extraction of oxidant-related compounds. This highlights the selective nature of extraction solvents and the dependence of measured parameters on overall extract composition [22].

Correlation analyses clearly demonstrated the biochemical relationships between antioxidant and oxidant parameters. The positive and significant correlations between TPC and TAS in both the ethanol and methanol extracts confirm that phenolic compounds are among the main determinants of total antioxidant capacity. Similar results have been demonstrated by Sicari [23], Condelli et al. [22], and Lammi et al. [38], who highlighted the strong correlation between total polyphenol content and antioxidant activity, as well as their contribution to oxidative stability in olive oil. Likewise, the positive relationship between TPC and DPPH RSA supports the role of phenolic compounds in free radical scavenging mechanisms [23]. In contrast, the negative correlations observed between TPC and TAS with OSI indicate that higher antioxidant capacity is associated with lower oxidative stress levels. These results are consistent with the results shown by De Bruno et al. [39], who reported that phenolic compounds enhance oxidative stability. Furthermore, Gorinstein et al. [4] and Güzel et al. [5] also found that higher TPC levels lead to increased antioxidant activity and that a linear relationship exists between phenolic content and antioxidant capacity.

The weak correlations between DPPH RSA and and oxidative stress parameters (TOS and OSI) suggest that DPPH RSA reflects only certain aspects of antioxidant activity and may not fully capture the overall antioxidant-oxidant status of the samples [40]. Therefore, the combined use of multiple analytical methods may provide a more comprehensive assessment of antioxidant and oxidative status. Comparative analyses showed that ethanol extracts exhibited higher TPC and DPPH RSA values, whereas methanol extracts showed higher TAS values, indicating that solvent polarity may influence the extraction of different antioxidant constituents. Ethanol extracts also exhibited higher TOS and OSI values, suggesting differences in the co-extraction of oxidant-related compounds. These findings are consistent with previous reports indicating that solvent polarity significantly affects extraction efficiency and phenolic composition [26].

Statistical analyses indicated substantial and statistically significant differences between solvents, particularly for DPPH RSA, TOS, and OSI. Although Bonferroni correction reduced the significance of TPC and TAS differences, the consistency of results under the FDR correction supports the robustness of the findings. Bland–Altman analysis confirmed systematic differences between extraction systems, with ethanol extracts generally yielding higher values for several parameters. In contrast, TAS exhibited only a small bias between solvents, indicating comparatively similar measurements across the two extraction methods [39].

PCA results revealed a multivariate structure in which antioxidant (TPC, TAS, DPPH RSA) and oxidant (TOS, OSI) parameters were clearly separated. The first two principal components (PC1 and PC2) explained 76.9% of the total variance (47.2% and 29.7%, respectively), indicating that the dataset can be effectively represented along these two axes.

The variables contributing most to the first principal component (PC1) were OSI and TOS, which showed a strong positive correlation. In contrast, TPC and TAS were negatively associated with this component. This distribution indicates that antioxidant capacity parameters (TPC, TAS, and DPPH RSA) are located on one side of the PC1 axis, whereas oxidative stress indicators are positioned on the opposite side. Accordingly, variables representing antioxidant capacity and oxidative stress parameters are clearly separated along this axis. OSI was identified as the most influential variable in PC1, explaining approximately 30% of the variance within this component.

On the PC2 axis, TOS (0.600) and DPPH RSA (0.475) exhibited high loadings. This suggests that oxidant load and radical scavenging activity share a common variation pattern along the PC2 axis. In particular, the positioning of DPPH RSA on this axis implies that this method may be sensitive to certain specific groups of antioxidant compounds independent of total phenolic content [40,41]. Similarly, the notable contribution of TOS to PC2 indicates that oxidant load includes additional sources of variation that cannot be explained solely by antioxidant capacity.

When the PCA score plot was examined, ethanol and methanol extracts showed a clear tendency to separate from each other. This separation indicates that the type of solvent used significantly influences the overall antioxidant and oxidant profile of the samples. In particular, the clustering of ethanol and methanol extracts along different axes supports the decisive role of the extraction solvent in determining the distribution of bioactive compounds. Overall, the PCA results demonstrate that the quality evaluation of olive oil samples can be more accurately achieved using a multivariate analytical approach rather than relying on a single parameter.

The radar plot results further support these findings, showing that 80:20 ethanol/water extracts exhibited higher TPC and DPPH RSA compared with 80:20 methanol/water extracts. In contrast, methanol extracts exhibited relatively higher TAS values, suggesting that solvent composition may differentially influence antioxidant-related parameters. Although methanol is generally considered an effective solvent for the extraction of phenolic compounds due to its higher polarity, the extraction efficiency depends not only on solvent polarity but also on the sample matrix, the chemical structure of phenolic compounds, and solvent–compound interactions [9]. Similarly, Do et al. [11] reported that ethanol can be more effective than methanol in extracting certain groups of phenolic compounds. Furthermore, Condelli et al. [22] found that different antioxidant assays may yield significantly variable results due to differences in reaction mechanisms and solvent systems. Such variations may become even more pronounced in complex matrices such as olive oil.

The observed differences between methanol and ethanol extracts may be associated with the selective extraction of phenolic compounds with different polarities and solvent–matrix interactions. However, individual phenolic compounds were not identified in the present study; therefore, the specific chemical constituents responsible for the differences between extraction systems could not be determined. In addition, since only methanol- and ethanol-based hydroalcoholic extraction systems were evaluated, the findings of this study cannot be directly extrapolated to other solvent systems with different physicochemical properties. Nevertheless, the results emphasize the important role of solvent polarity and solvent composition in influencing extraction efficiency and antioxidant-related parameters in EVOOs.

## 5. Conclusions

The findings of this study demonstrate that the antioxidant and oxidative stress profile of olive oil is strongly influenced not only by intrinsic sample characteristics but also by the extraction method used. This highlights the importance of standardizing analytical procedures in olive oil quality assessment and emphasizes the need to evaluate multiple parameters simultaneously for accurate interpretation of antioxidant capacity.

## Figures and Tables

**Table 1:** Distribution characteristics of antioxidant and oxidant parameters in olive oil samples according to extraction methods.

Parameter	Solvent Type	Skewness	Kurtosis	Shapiro–Wilk, (*p*-Value)
TPC	Ethanol	0.50	−0.10	0.075
	Methanol	1.08	0.88	<0.001
DPPH RSA	Ethanol	0.19	−0.99	0.046
	Methanol	0.65	−0.26	0.006
TAS	Ethanol	0.37	−0.38	0.347
	Methanol	1.71	3.78	<0.001
TOS	Ethanol	0.67	−0.67	0.001
	Methanol	0.66	−1.10	<0.001
OSI	Ethanol	0.75	−0.59	<0.001
	Methanol	1.12	0.69	<0.001

DPPH RSA: 2,2-diphenyl-1-picrylhydrazyl radical scavenging activity; TPC: total phenolic content; TOS: total oxidant status; TAS: total antioxidant status; OSI: oxidative stress index.

**Table 2:** Descriptive statistics of antioxidant and oxidant parameters in olive oil samples determined by ethanol and methanol extraction methods.

Parameter/ Solvent Type	Mean	SEM	Min	10th perc	25th perc	Median	75th perc	90th perc	Max
TPC, mg GAE/kg									
Ethanol	138.1	59.2	50.2	56.8	88.2	139.1	166.4	225.4	297.0
Methanol	123.0	73.0	10.1	50.4	57.3	114.1	160.6	236.8	341.4
DPPH RSA, %									
Ethanol	24.48	7.15	13.44	14.40	18.71	23.99	30.04	34.09	38.77
Methanol	15.42	3.98	10.18	10.76	12.08	15.06	18.18	21.37	26.14
TAS, mmol TE/kg									
Ethanol	2.8	1.0	1.0	1.6	2.0	2.8	3.5	4.0	5.3
Methanol	3.2	1.4	1.1	1.8	2.4	2.9	3.6	4.6	7.8
TOS, µmol H_2_O_2_ Eq/kg									
Ethanol	84.6	42.0	23.5	39.4	51.7	76.7	106.7	161.2	168.8
Methanol	70.5	53.5	9.5	17.5	25.3	45.5	122.4	161.7	169.1
OSI									
Ethanol	3.51	2.23	0.61	1.20	1.64	2.83	5.25	7.10	8.42
Methanol	2.51	2.08	0.28	0.59	0.74	1.59	4.20	5.58	8.71

*n* = 55; data are presented as mean ± SEM (standard error of mean) along with percentile distributions (10–90%); GAE: gallic acid equivalent; TPC: total phenolic content; DPPH RSA: 2,2-diphenyl-1-picrylhydrazyl radical scavenging activity; TE: Trolox equivalent; TAS: total antioxidant status; TOS: total oxidant status; H_2_O_2_ Eq: hydrogen peroxide equivalent; OSI: oxidative stress index.

**Table 3:** Spearman correlation coefficients (r) between antioxidant and oxidative stress parameters in ethanol and methanol extracts of olive oil samples.

		Ethanol				Methanol			
Parameter		DPPH	TAS	TOS	OSI	DPPH	TAS	TOS	OSI
TPC	r	0.48	0.64	−0.08	−0.39	0.57	0.66	−0.17	−0.46
	*p*-value	<0.001	<0.001	0.579	0.003	<0.001	<0.001	0.213	<0.001
DPPH	r		0.44	−0.03	−0.26		0.47	0.00	−0.22
	*p*-value		0.001	0.802	0.051		<0.001	0.997	0.104
TAS	r			−0.24	−0.71			0.07	−0.35
	*p*-value			0.075	<0.001			0.618	0.009
TOS	r				0.84				0.90
	*p*-value				<0.001				<0.001

Values are presented as Spearman correlation coefficients (r) and significance levels (*p*). Statistical significance was set at *p* < 0.05. DPPH: 2,2-diphenyl-1-picrylhydrazyl; TPC: total phenolic content; TOS: total oxidant status; TAS: total antioxidant status; OSI: oxidative stress index.

**Table 4:** Spearman correlation coefficients of antioxidant and oxidant parameters between extraction solvents.

Parameter	Spearman Correlation Coefficient (r)	*p*-Value
TPC	0.735	<0.001
DPPH	0.518	<0.001
TAS	0.579	<0.001
TOS	0.706	<0.001
OSI	0.807	<0.001

DPPH: 2,2-diphenyl-1-picrylhydrazyl; TPC: total phenolic content; TOS: total oxidant status; TAS: total antioxidant status; OSI: oxidative stress index.

**Table 5:** Comparison of antioxidant and oxidant parameters between ethanol and methanol extracts (Wilcoxon signed-rank test).

Parameter	Median (Ethanol)	Median (Methanol)	Median Difference (Ethanol-Methanol)	% Change	Effect Size (r)	Wilcoxon *p*-Value	Bonferroni-Adjusted *p*-Value	FDR-Adjusted *p*-Value
TPC	139.15	114.14	13.22	17.97	−0.294	0.028	0.140	0.034
DPPH RSA	23.99	15.06	8.46	37.25	−0.851	<0.001	<0.001	<0.001
TAS	2.80	2.91	−0.33	−4.01	−0.283	0.034	0.171	0.034
TOS	76.71	45.55	14.08	40.62	−0.484	<0.001	<0.001	<0.001
OSI	2.83	1.59	0.76	43.87	−0.629	<0.001	<0.001	<0.001

Data were evaluated using median values. Effect size (r) was calculated based on the z-value of the Wilcoxon test. For multiple comparisons, Bonferroni and Benjamini–Hochberg (FDR) corrections were applied. Statistical significance was accepted at *p* < 0.05. DPPH RSA: 2,2-diphenyl-1-picrylhydrazyl radical scavenging activity; TPC: total phenolic content; TOS: total oxidant status; TAS: total antioxidant status; OSI: oxidative stress index.

**Table 6:** Bland–Altman analysis of agreement between ethanol and methanol extracts (bias and 95% limits of agreement).

Parameter	Bias (Mean Difference)	LoA (Lower, Upper Limits of Agreement)
TPC	−15.04	−115.20, 85.11
DPPH	−9.06	−21.21, 3.09
TAS	0.37	−1.80, 2.53
TOS	−14.13	−82.26, 54.00
OSI	−1.00	−3.80, 1.79

Bias: mean difference (ethanol—methanol); LoA (limits of agreement): 95% limits of agreement (±1.96 SD). DPPH: 2,2-diphenyl-1-picrylhydrazyl; TPC: total phenolic content; TOS: total oxidant status; TAS: total antioxidant status; OSI: oxidative stress index.

**Table 7:** Principal component analysis (PCA) loading matrix and contribution percentages of antioxidant and oxidant parameters.

Parameter	Loading Matrix		Contribution Percentages	
	PC1 (47.2%)	PC2 (29.7%)	PC1	PC2
TPC	−0.491	0.404	0.299	0.167
DPPH	−0.257	0.475	0.241	0.163
TAS	−0.487	0.290	0.237	0.084
TOS	0.396	0.600	0.157	0.360
OSI	0.547	0.409	0.066	0.226

PC1 and PC2 explain 47.2% and 29.7% of the total variance, respectively. DPPH: 2,2-diphenyl-1-picrylhydrazyl; TPC: total phenolic content; TOS: total oxidant status; TAS: total antioxidant status; OSI: oxidative stress index.

## Data Availability

The original contributions presented in this study are included in the article. Further inquiries can be directed to the corresponding author.

## Abbreviations

The following abbreviations are used in this manuscript:

DPPH	2,2-diphenyl-1-picrylhydrazyl
EVOO	Extra virgin olive oil
OSI	Oxidative stress index
PCA	Principal component analysis
TAC	Total antioxidant capacity
TAS	Total antioxidant status
TOS	Total oxidant status
TPC	Total phenolic content
